# Effects of vestibular neurectomy and neural compensation on head movements in patients undergoing vestibular schwannoma resection

**DOI:** 10.1038/s41598-020-79756-3

**Published:** 2021-01-12

**Authors:** Omid A. Zobeiri, Gavin M. Mischler, Susan A. King, Richard F. Lewis, Kathleen E. Cullen

**Affiliations:** 1 Department of Biomedical Engineering, McGill University, Montreal, QC Canada; 2 Department of Biomedical Engineering, Johns Hopkins University, Baltimore, USA; 3 Jenks Vestibular Physiology Laboratory, Massachusetts Eye and Ear Infirmary, Boston, MA USA; 4 Departments of Otolaryngology and Neurology, Harvard Medical School, Boston, MA USA; 5 Department of Otolaryngology-Head and Neck Surgery, Johns Hopkins University School of Medicine, Baltimore, USA; 6 Department of Neuroscience, Johns Hopkins University School of Medicine, Baltimore, USA; 7 Kavli Neuroscience Discovery Institute, Johns Hopkins University, Baltimore, USA

**Keywords:** Neural encoding, Sensory processing, Reflexes

## Abstract

The vestibular system is vital for maintaining balance and stabilizing gaze and vestibular damage causes impaired postural and gaze control. Here we examined the effects of vestibular loss and subsequent compensation on head motion kinematics during voluntary behavior. Head movements were measured in vestibular schwannoma patients before, and then 6 weeks and 6 months after surgical tumor removal, requiring sectioning of the involved vestibular nerve (vestibular neurectomy). Head movements were recorded in six dimensions using a small head-mounted sensor while patients performed the Functional Gait Assessment (FGA). Kinematic measures differed between patients (at all three time points) and normal subjects on several challenging FGA tasks, indicating that vestibular damage (caused by the tumor or neurectomy) alters head movements in a manner that is not normalized by central compensation. Kinematics measured at different time points relative to vestibular neurectomy differed substantially between pre-operative and 6-week post-operative states but changed little between 6-week and > 6-month post-operative states, demonstrating that compensation affecting head kinematics is relatively rapid. Our results indicate that quantifying head kinematics during self-generated gait tasks provides valuable information about vestibular damage and compensation, suggesting that early changes in patient head motion strategy may be maladaptive for long-term vestibular compensation.

## Introduction

The vestibular system contributes to gaze stabilization, postural stability, and the perception of head motion and orientation. Following unilateral peripheral vestibular loss patients experience impaired vision and imbalance (e.g.,^1,2^), as well as aberrant motion and orientation perception (e.g.,^3^). These symptoms can be debilitating, making it difficult for patients to perform their normal daily activities. Because vestibular damage is usually accessed by measuring vestibular-mediated eye movements (e.g., during caloric or rotational stimulation) and postural stability (reviewed in^4–7^), its effects on head motion have been relatively overlooked. Head movement kinematics, however, may be particularly.

informative about the *integrity of the peripheral* vestibular system and the state of *central compensation* because head motion directly activates the vestibular sensors located within the skull. To investigate the hypothesis that head kinematics are significantly altered as a result of peripheral vestibular loss during voluntary behavior, here we measured and quantified head motion in patients who transitioned through a series of vestibular states characterized by different levels of peripheral damage and central compensation.

We chose to use the ten tasks that constitute the Functional Gait Assessment (FGA), since this allowed us to compare our quantitative analysis of head kinematics to the FGA scores that are commonly used in the clinic to assess functional capacity and fall risk in patients with vestibular damage. Notably, FGA scores are inherently *subjective*, as they are scored by a clinician observing the patient. In addition, considerable *information* is lost by FGA scores since each task is assigned an integer value between 0 and 3, even though kinematics during these tasks are distributed along a continuum^8,9^. Furthermore, the FGA test battery is *time-consuming* and the most valuable type of information that can be culled from these gait tests has not been elucidated. Measurements of head motion kinematics during the FGA in vestibular patients, therefore, offer numerous potential advantages, since these measures are objective and quantified, and can be examined to define the most efficient gait tasks and kinematic measures that identify the impairments caused by vestibular damage and the improvements due to central compensation.

Thus to determine how head kinematics are altered as a result of peripheral vestibular loss during voluntary behavior and, in turn, provide new insight into potential constraints on the neural mechanisms that mediate vestibular compensation, we quantified performance during each of the ten tasks of the FGA. Unilateral vestibular schwannoma (VS) patients were tested before and following (6 weeks and > 6 months) surgical resection of the tumor, which required complete sectioning of the vestibular nerve. We first recorded head movements in all six dimensions of motion (three rotational, three translational axes) using a micro-electromechanical system (MEMS) sensor. Next using objective quantification methods, we then computed measures of variability and range of motion for each axis, as well as gait speed and symmetry, during each task. We compared these measures for our patient group across each of the three time points and then contrasted these with measures obtained from age-matched healthy control subjects. We found that specific kinematic measurements during the most challenging FGA tasks segregated pre-op, sub-acute (6 week) post-op, and chronic (6 month) post-op VS patients from normal control subjects, and that the effects of vestibular neurectomy and compensation could be identified in VS patients by comparing head kinematics at these three different time points. Notably, during these tasks, patients displayed marked changes in the range and variability of head movements, principally along the vertical axis, as well as time for task completion. Taken together our results show that head kinematics are significantly altered in patients, even prior to surgery as a result of the presence of the tumor, suggesting that early changes in head motion strategy set the upper limit for recovery of normative head motion in these patients. Furthermore, our results provide an example for advancing the development of shorter, more refined, and more predictive tests of clinical status.

## Results

**Figure 1:**
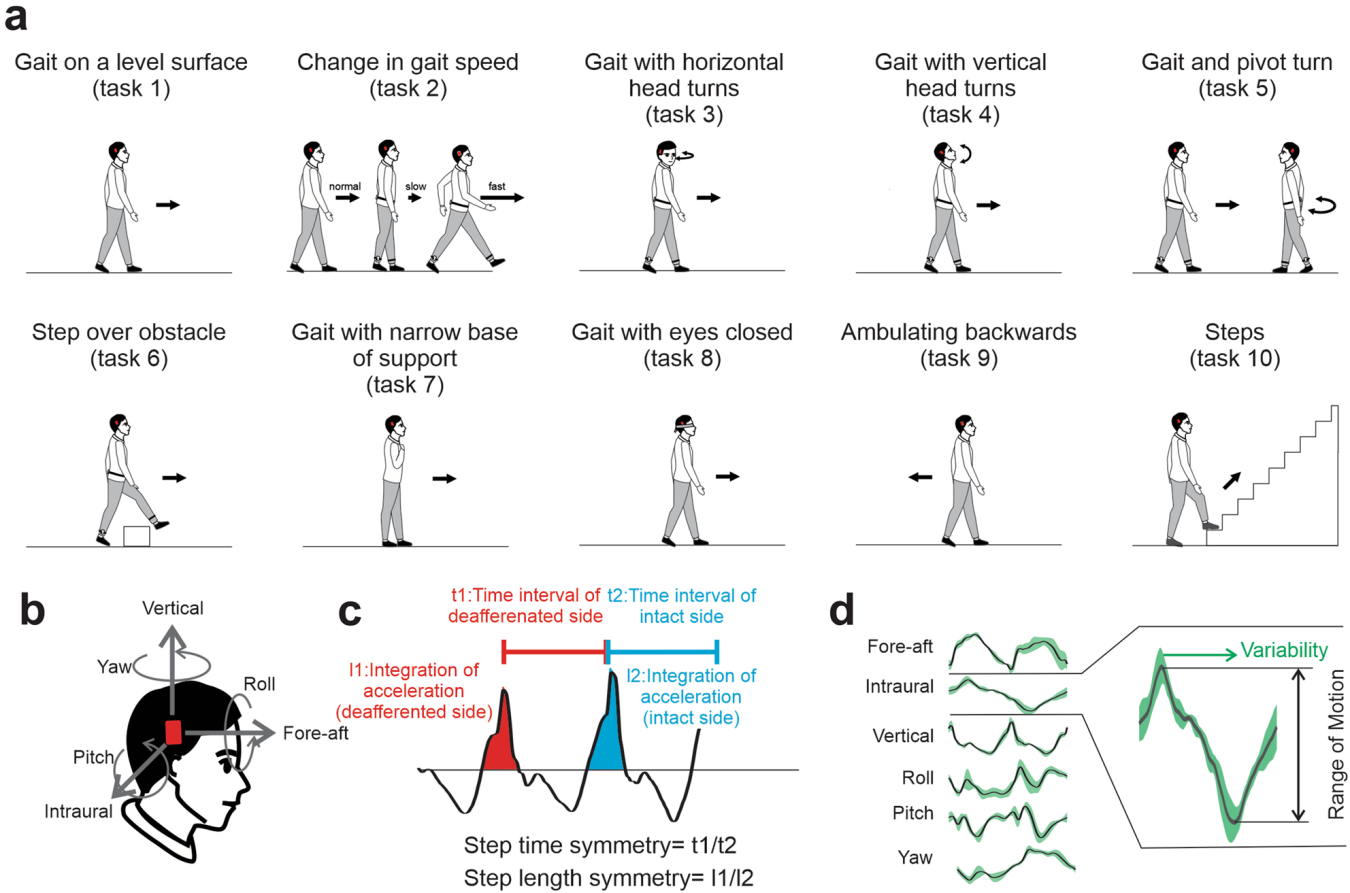
(**a**) Schematic of 10 Functional Gait Assessment (FGA) tasks: (1) ‘gait on a level surface’, (2) ‘change in gait speed’, (3) ‘gait with horizontal head turns’, (4) ‘gait with vertical head turns’, (5) ‘gait and pivot turn’, (6) ‘step over obstacle’, (7) ‘gait with narrow base of support’, (8) ‘gait with eyes closed’, (9) ‘ambulating backwards’, and (10) ‘steps’ (**b**) Subjects’ head motion during FGA was recorded using a MEMS sensor attached to their head which comprises three linear accelerometers (recording linear accelerations along the fore-aft, interaural, and vertical axes) and three gyroscopes (recording angular velocity about pitch, roll, and yaw). (**c**) Asymmetry measures defined as 1) ‘step time asymmetry’, estimated from the time intervals between vertical acceleration peaks for each leg's heel strike was calculated and 2) ‘step length asymmetry’, estimated as the integration of the positive vertical accelerations following heel strike of each leg was calculated. (**d**) Variability and range of motion were quantified for each of the six axes of head motion as the average of the standard deviation and magnitude of the signal across gait cycles in each task.

**Figure 2:**
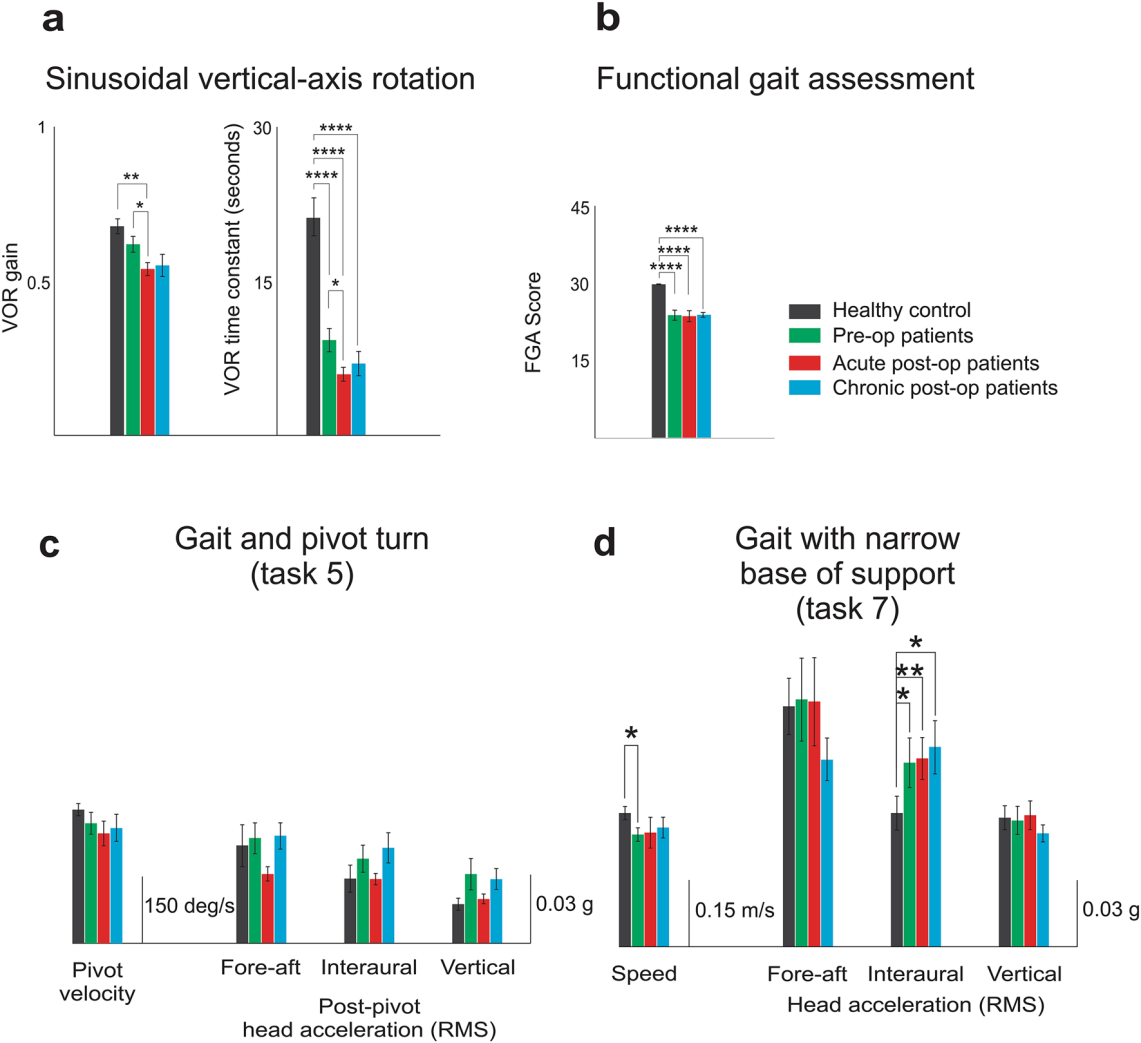
(**a**) Vestibular function of patients assessed via VOR gain (left panel) and time constant (right panel). (**b**) Functional capacity of patients assessed by FGA scores. (**c**) Objective kinematic measures during ‘gait and pivot turn’ (task 5): pivot velocity and root mean square of post-pivot head acceleration for all three axes. (**d**) Objective kinematic measures during ‘gait with the narrow base of support’ (task 7): completion speed and root mean square of head acceleration for all three axes.

Twenty-three patients with unilateral VS and twenty-one normal control subjects were included in the study (see Methods). We first assessed vestibular function and capacity using VOR testing and standard scoring on the FGA (described in Table 1 and shown schematically in Fig. 1a), respectively. Figure 2a illustrates the distribution of VOR time constants and gains measured for patients before surgery (“pre-op”) and then 6 weeks after surgery (“sub-acute post-op”) and > 6 months after surgery (“chronic post-op”). Comparison across populations and time points revealed lower time constants for patients in each state, relative to controls (*p* < 0.0001). Following surgery, patients showed a significant decrease in time constant relative to pre-op values (*p* < 0.05) which returned to values not significantly different than those recorded in the pre-op or sub-acute values (*p* > 0.05). In contrast, analysis of VOR gains revealed no difference between healthy controls and pre-op patients (*p* > 0.05), but patients then showed significantly lower gains after surgery (i.e., sub-acute state, *p* < 0.05) that then returned to pre-op values in the chronic state (*p* > 0.05). Each subject's functional capacity was also scored using the conventional FGA test evaluated by a clinician (an integer value between 0 and 3 was assigned to each of the 10 tasks). Figure 2b demonstrates that the patients’ FGA scores were significantly worse than those of healthy controls at each of the 3 time points (i.e., pre-op, sub-acute, and chronic, *p* < 0.0001). We further found that FGA scores did not differ for patients when compared across each of these 3 measured time points (*p* > 0.05). Specifically, patient scores were comparable in the sub-acute compared to the pre-op state and then remained unchanged even after 6 months.

**Table 1:** List of the kinematic measures used in this study.

		FGA Objective Measures
**Test**	**Measure**	**Description**
Tasks 1–4,6,8–10	Gait Speed	**Speed** (distance/duration)
		**Steps/Sec**
	Gait asymmetry	**Step length asymmetry:** The ratio of integration of the positive vertical accelerations following heel strike of each leg (deafferenated side/ intact side)
		**Step time asymmetry:** The ratio of time intervals between vertical acceleration peaks (deafferenated side/ intact side)
	Gait variability	Average of the standard deviation of head movement across gait cycles for along each of the three dimensions of rotation and translation
		**Fore-aft** (translation)
		**Interaural** (translation)
		**Vertical** (translation)
		**Roll** (rotation)
		**Pitch** (rotation)
		**Yaw **(rotation)
	Range of motion	Average of the range of head movement across gait cycles along each of the three dimensions of rotation and translation:
		**Fore-aft **(translation)
		**Interaural** (translation)
		**Vertical** (translation)
		**Roll** (rotation)
		**Pitch** (rotation)
		**Yaw **(rotation)
Task 5	Pivot velocity	The peak of yaw angular velocity during the pivot
	Post-pivot head motion sway	Post-pivot head motion sway, calculated as the root mean square of linear accelerations in the horizontal plane within two seconds after the end of the turn
Task 7	Speed	The distance divided by the time of completion of the task
	Head motion sway	Root mean square of linear accelerations in the horizontals plane during the task

### Head movement kinematics are abnormal in VS patients

To quantify subject performance during each FGA task, we first quantified head movement kinematics (see “Methods”) during the two tasks where individual gait cycles could not be reliably identified, the ‘gait with pivot turn (task 5)’ and ‘gait with narrow base of support’ (task 7). Our analysis of ‘Gait with pivot turn’ (task 5) did not reveal any significant differences in head kinematics between different groups. Specifically, both peak pivot velocity and post-pivot head acceleration were comparable to healthy controls for each patient at all three time points (Fig. 2c; *p* > 0.05). In contrast, during ‘gait with narrow base of support’ (task 7), our analysis revealed increased instability along the interaural axis (i.e., increased lateral sway) in all patient groups compared to healthy controls (Fig. 2d, *p < 0.05, **p < 0.01). We also found that pre-op (but not post-op) patients were slower than normal in completing the task (Fig. 2d, Speed measure).

**Figure 3:**
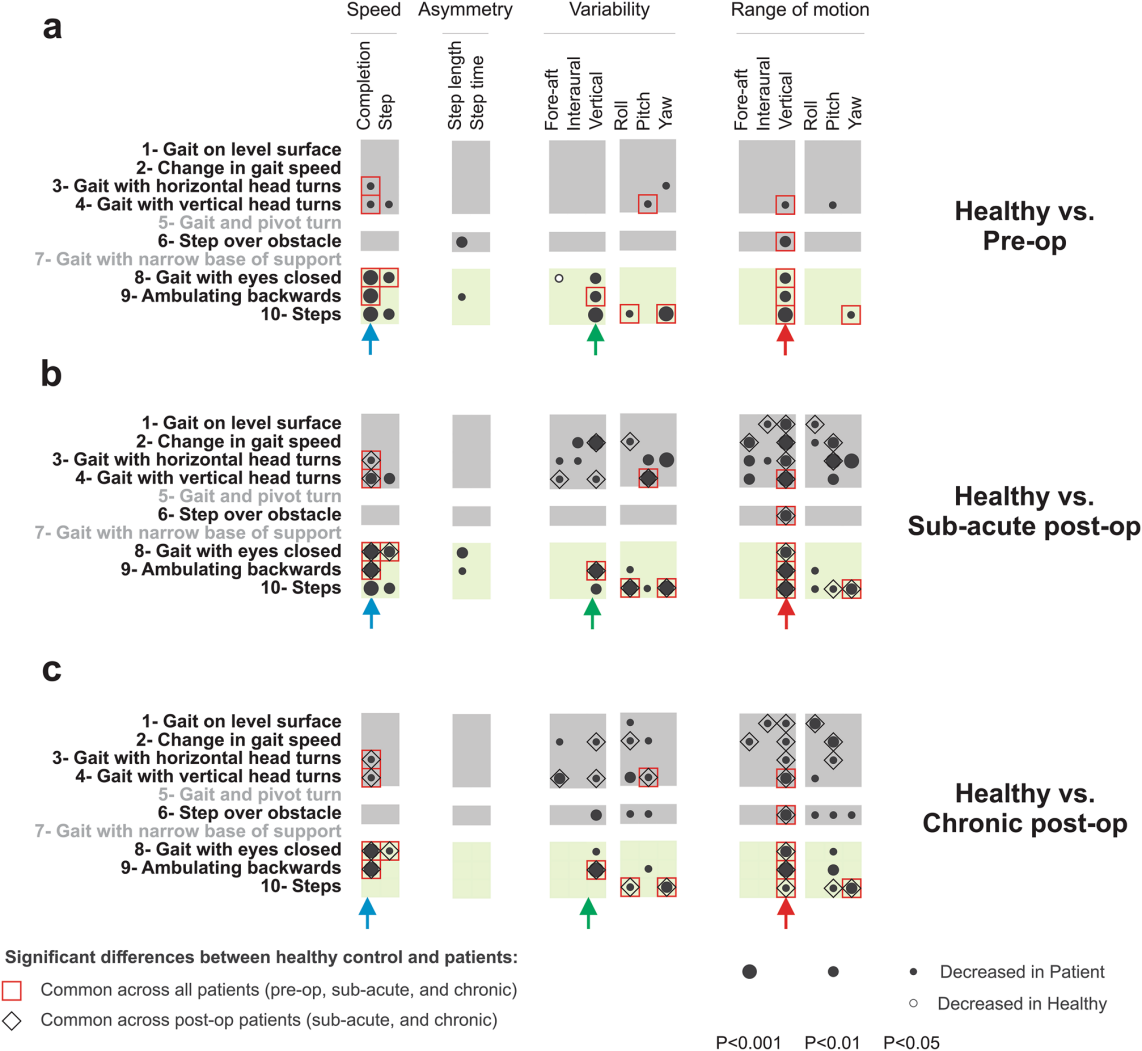
Comparison of objective head movement kinematics between (**a**) pre-op (**b**) sub-acute post-op (**c**) chronic post-op patients and healthy controls during FGA tasks (1–4,6,8–10). (**a**–**c**) Each row corresponds to one of the FGA tasks, while columns correspond to kinematic measures that are associated with one of four main categories; (1) speed, (2) asymmetry, (3) variability, and (4) range of motion. The circles demonstrate the kinematic measures in each task that distinguish patients from healthy controls. Dark circles show decreased measure in patients, while the white circle means that measure was decreased in the healthy group. The radius of the circle indicates the level of significance of the difference between the two groups. Open red squares denote the differences with the healthy subjects that are common in patients across all time points. Open black diamonds show the differences that are common only across two post-op time points. Blue, green, and red arrows highlight specific kinematic measures that were most informative across all FGA tasks.

We next focused our analysis on the remaining 8 tasks of the FGA for which we could reliably detect individual gait cycles (i.e., tasks 1–4,6,8–10). After identifying gait cycles within each of these 8 tasks, we quantified head movement kinematics (see “Methods”) for the VS patients at each time point and for healthy controls. Figure 3 illustrates which of the kinematic measures were significantly different for pre-op (Fig. 3a), sub-acute post-op (Fig. 3b), and chronic post-op patients (Fig. 3c) when compared to healthy controls, for each task. Black and white circles indicate measures for which we observed significant decrease and increase in patients’ kinematic, respectively, relative to those of healthy controls. Notably, each column represents a kinematic measure associated with one of four main categories: (1) speed, (2) asymmetry, (3) variability, and (4) range of motion.

Figure 3a compares the head kinematics measures for *pre-op patients and healthy controls*. Our assessment of these measures revealed that three of the *more challenging FGA* tasks were the most informative, namely: ‘gait with eyes closed’ (task 8), ‘ambulating backwards’ (task 9), and ‘steps’ (task 10) (Fig. 3a, bottom green-shaded rows). Specifically, during these three tasks, both the *variability and range of vertical head acceleration* were reduced in pre-op patients relative to controls (Fig. 3a, green and red arrows, respectively; *p* < 0.01). We also found that *pre-op patients were slower* during each of these three tasks, as quantified by the increase in time required for task completion (*p* < 0.001, blue arrow). In contrast, we did not observe consistent differences between our pre-op patients and healthy controls during the less challenging tasks—for example, the ‘gait with level surface’ (task 1) (Fig. 3a, top gray-shaded row).

*Sub-acute post-op VS patients* (Fig. 3b) also showed significant differences from normal subjects. Like the pre-op VS patients, abnormalities in the sub-acute post-op patients were most marked for the vertical linear axis and the primary abnormal motion characteristics remained decreased variability and range of motion. However, compared to pre-op patients, sub-acute post-op patients showed abnormalities across a wider range of FGA tasks (compare Fig. 3a,b), even including some of the less challenging tasks (grey shaded rows). Finally, similar to pre-op patients, sub-acute post-op patients took longer to complete the majority (5/8) of tasks (*p* < 0.05, denoted by the blue arrow) 6 weeks after surgery.

Finally, when we tested the same VS patients in the chronic post-op state (Fig. 3c), we found that many of the changes observed relative to healthy controls 6 weeks following surgery persisted > 6 months after surgery (common changes denoted by open black diamonds in Fig. 3b,c). Specifically, the range of vertical head accelerations remained reduced relative to healthy controls across all tasks (Fig. 3c; red arrow). Likewise, patients showed decreased variability of vertical head acceleration across (5/8) tasks (green arrow), and also took longer to complete many (4/8) tasks (blue arrow) 6 weeks after surgery (sub-acute). Overall, it is noteworthy that 6 months following surgery patients showed fewer significant differences relative to healthy controls than they did 6 weeks following surgery (compare Fig. 3b,c). Furthermore, comparison of patient performance at each of the three time points (i.e., pre-op, sub-acute post-op, and chronic post-op) revealed significant differences that were common for patients when compared to healthy controls (denoted by open red squares, Fig. 3a–c). Specifically, a significant decrease in speed and range of vertical head acceleration in many FGA tasks persisted from pre-op to the chronic post-op testing.

### Head movement kinematics are altered in VS patients 6 weeks after surgery relative to preoperative measures

**Figure 4:**
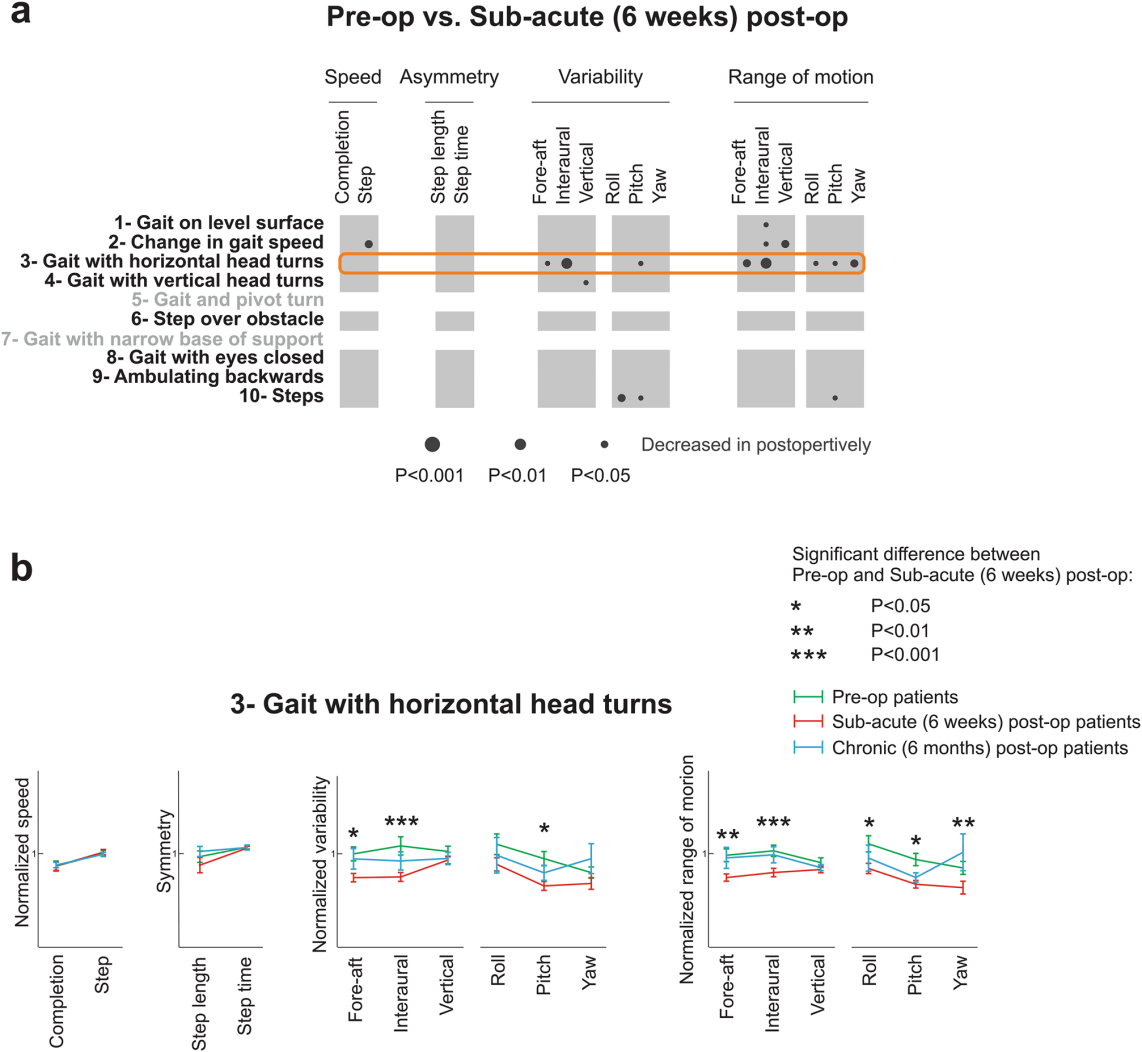
Comparison of objective head movement kinematics of patients 6 weeks following the surgery with their pre-op state. (**a**) Each column corresponds to a kinematic measure, while rows indicated FGA tasks. Dark circles show decreased measure 6 weeks after the surgery. The radius of the circle indicates the level of significance of the difference between the two groups. Orange box indicates the FGA task in which patients altered their kinematics the most from pre-op and sub-acute patients (**b**) The normalized value of all kinematic measures for patients across all time points during more informative tasks of the FGA: ‘gait with horizontal head turns’ (task 3). Green, red, and blue lines correspond to pre-op, sub-acute post-op, and chronic post-op patients. Asterisks denote the kinematic measures that were significantly different between pre-op and sub-acute post-op patients.

To directly evaluate the effects of complete unilateral vestibular deafferentation, we next compared head kinematics in VS patients when they were in the pre-operative state with their kinematics in the sub-acute (6 weeks) post-op state. It is important to note, however, that patient function at sub-acute post-op reflects the loss of vestibular function due to deafferentation combined with the central compensation that occurred over this sub-acute time frame. As indicated by black circles in Fig. 4a,b number of kinematic measures showed significant changes at sub-acute post-op relative to the pre-op state. These differences were most notable for the ‘gait with horizontal head turns’ (task 3) (denoted by the orange box), during which subjects were asked to make voluntary side-to-side head movements. Differences in measures for pre-op and sub-acute post-op patients during this task are shown in Fig. 4a (green vs. red lines, respectively). Specifically, following surgery patients showed a reduction in the translational and rotational range of motion in all six dimensions except vertical acceleration (*p* < 0.05, Fig. 4a right six columns). Further, sub-acute patients also showed a reduction in the variability of their translational acceleration in the horizontal plane (i.e., fore-aft and interaural) as well as in the variability of their angular pitch velocity (*p* < 0.05). In addition, 6 weeks following surgery patients showed a decrease in their pitch and roll rotational variabilities during the ‘steps’ (task 10) (Fig. 4a, bottom row) relative to their preoperative performance (*p* < 0.05). Moreover, during the ‘change in gait speed’ (task 2) (Fig. 4a, second row), they showed a reduction in the range of their vertical head acceleration as well as the number of steps/second (*p* < 0.01).

### Head kinematics are comparable for sub-acute and chronic VS patients

**Figure 5:**
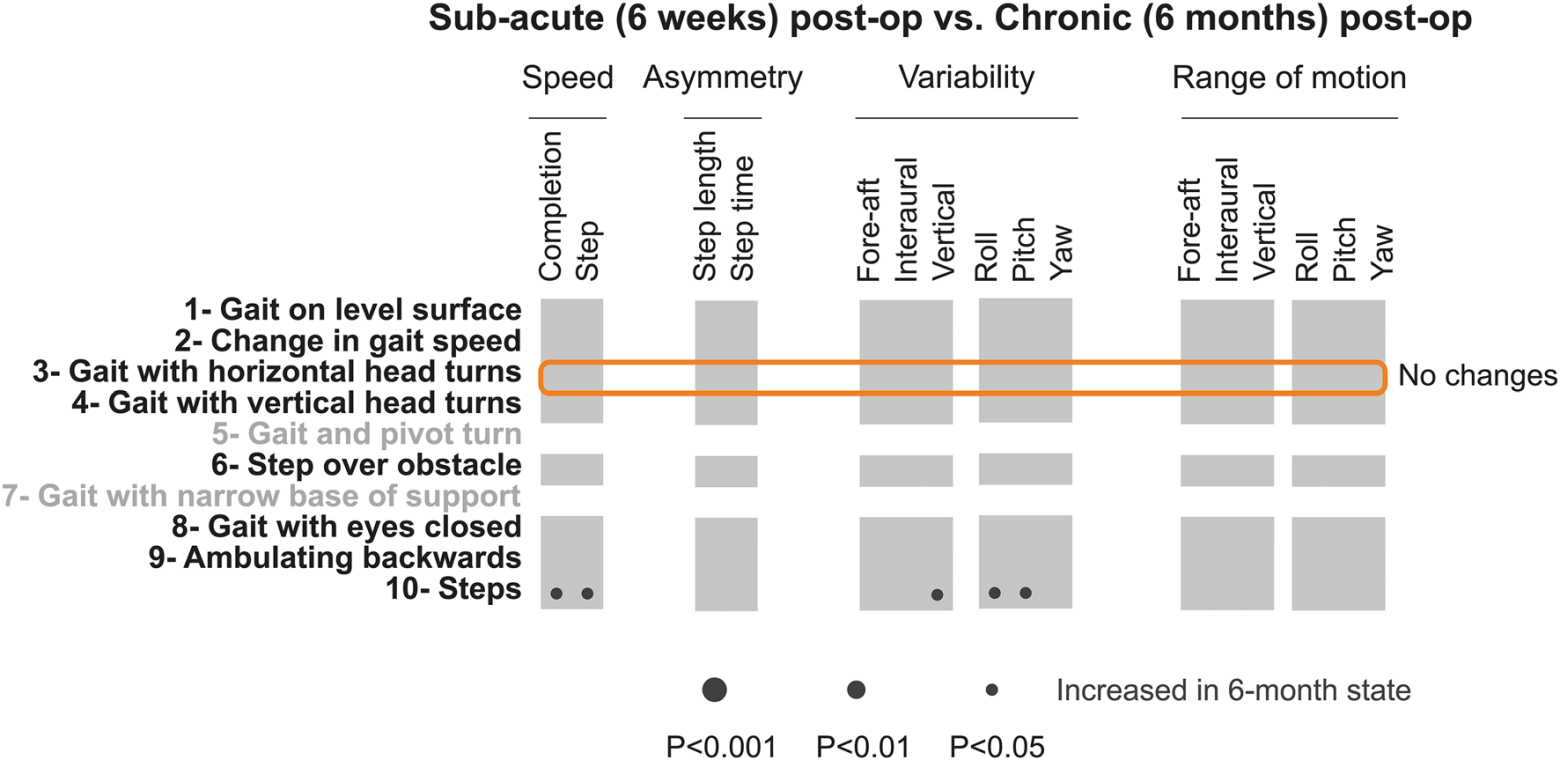
Comparison of objective head movement kinematics of sub-acute and chronic post-op patients. Each column corresponds to a kinematic measure, while rows indicated FGA tasks. Dark circles show increased measure > 6 months after the surgery. The radius of the circle indicates the level of significance of the difference between the two groups. Orange box indicates the FGA task in which patients altered their kinematics the most from pre-op and sub-acute post-op patients.

To assess whether any improvement occurred following the first 6 weeks of postoperative recovery, we compared the head kinematics during the same 8 tasks of the FGA for which we could reliably detect individual gait cycles for VS patients tested 6 weeks and > 6 months after surgery (Fig. 5). Surprisingly, our analysis revealed only a few differences in kinematics (denoted by the lack of circles), suggesting that compensation was largely complete by 6 weeks. For example, as shown in Fig. 5, we saw no significant change for the ‘gait with horizontal head turn task’ (task 3) (orange box). This is noteworthy since patients showed reduced variability and range of motion from pre-op to sub-acute post-op state (see Fig. 4a). However, we did not observe a subsequent improvement (increase) from 6 weeks to 6 months (Fig. 4b, blue vs. red bars). Notably, however, we did observe a significant increase in our two speed measures during ‘steps’ (task 10) (Fig. 5; *p* < 0.01; black circles, bottom row), suggesting that the patients’ performance of this task continued to improve after 6 weeks.

### Head movement kinematics were comparable in VS patients before and > 6 months after surgery

**Figure 6:**
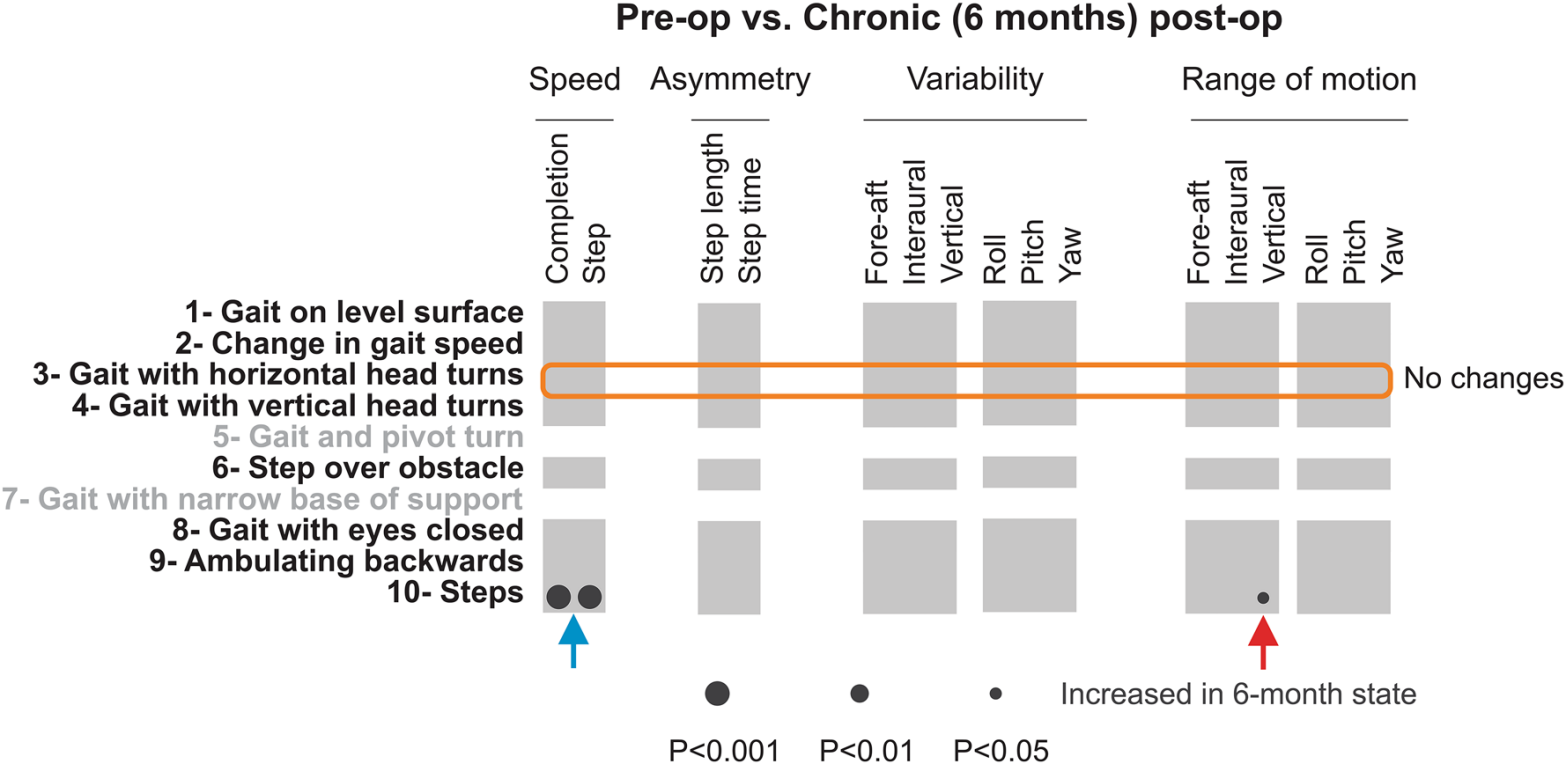
Comparison of objective head movement kinematics of pre-op and chronic post-op. Each column corresponds to a kinematic measure, while rows indicated FGA tasks. Dark circles show increased measure > 6 months after the surgery. The radius of the circle indicates the level of significance of the difference between the two groups. Orange box indicates the FGA task in which patients altered their kinematics the most from pre-op and chronic post-op patients. Blue and red arrows indicate specific kinematic measures, which were significantly changed from pre-op to chronic post-op states during the “steps” task.

Finally, we compared the kinematic measures of patients > 6 months following surgery with those of these same patients before surgery to assess changes in their kinematics relative to the pre-op state. This comparison is illustrated in Fig. 6, again for the 8 tasks of the FGA for which we could reliably detect individual gait cycles. Notably, we found that overall the kinematics of patients 6 months after surgery were largely comparable to their kinematics before surgery. Interestingly, for the ’gait with horizontal head turns’ (task 3) (Fig. 6, orange box) in which patients showed most significant changes 6 weeks following the surgery (see Fig. 4), we found no significant differences between kinematic measures after 6 months (Fig. 4b, green vs blue lines). Thus, during this task, patients actually increased the magnitude and variability of their head movements from their sub-acute post-op states to measures comparable with their own pre-op state. However, as discussed above in the previous section, these changes were not significant when directly compared to kinematics measured in the sub-acute post-op state. The black circles in the bottom row of Fig. 6 further indicate two significant differences between pre-op and chronic post-op state – both during the ’steps’ (task 10). Specifically, patients demonstrated an increase in both of our speed measures in the chronic post-op state (*p* < 0.01; Fig. 6, blue arrow). In addition, the range of their vertical head acceleration increased at the chronic post-op state (*p* < 0.05, red arrow). Taken together, these results suggest that the remaining effects of vestibular nerve deafferentation were partially, but not completely, compensated over the interval spanning 6 weeks to 6 months following surgery.

### Quantifying the global change in kinematics in VS patients before and after vestibular neurectomy based on the most informative head motion parameters

**Figure 7:**
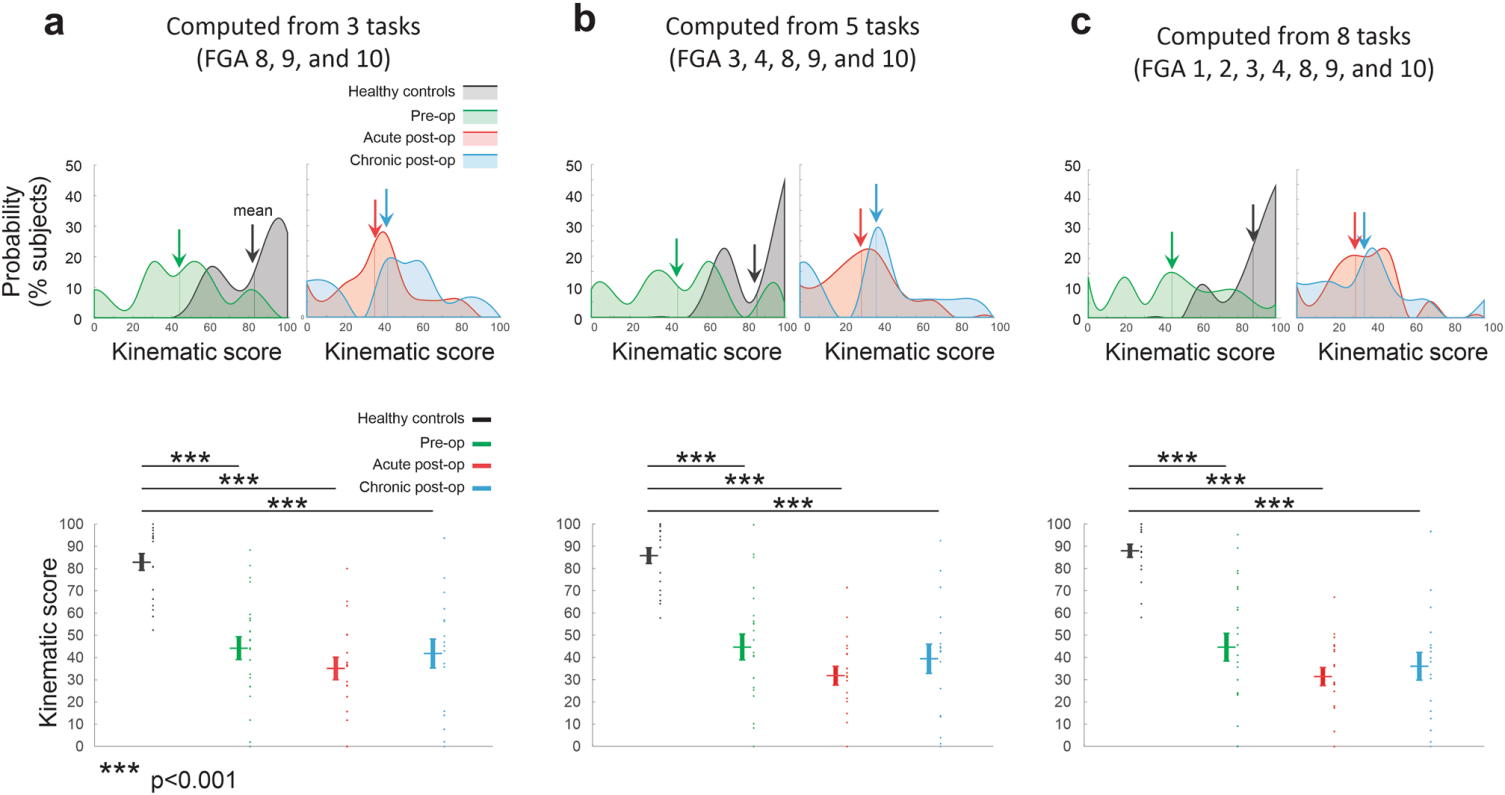
Comparison kinematic scores computed for (a) the 3 most challenging FGA tasks (FGA 8, 9, and 10), (b) 5 FGA tasks for which significant differences were typically observed between patients and controls (FGA 3, 4, 8, 9, and 10), and (c) across all 8 tasks for which gait cycles were extracted (FGA 1–4, 8, 9, and 10). (a-c) *Top-left:* Probability distributions of the kinematic scores computed for healthy controls (black), pre-op patients (green). Arrows indicate the average values. *Top-right:* Probability distributions of the kinematic scores computed for acute post-op (red), and chronic post-op patients (blue). *Bottom:* Comparison of the kinematic scores of healthy controls versus unilateral vestibular patients. Vertical lines correspond to mean ± SEM of the kinematic score for each group, while the kinematic score for individual subjects are illustrated as points. Asterisks denote significant difference between healthy controls and patients (***p < 0.001).

Our results demonstrate that measuring head movement kinematics – in particular the range and variability of vertical motion during more challenging FGA tasks—provides valuable information about vestibular damage and compensation. These results raise the question of whether it is feasible to collapse the wide range of measures we made into a global kinematic score that would be useful to clinicians who are evaluating VS patients before or after tumor resection. To this end, we computed a single score based on the three kinematic measures that were most informative in our results (i.e., task completion speed, vertical head acceleration variability, and vertical head acceleration range of motion, Fig. 3). We compared this score when it was computed for (1) only the 3 most challenging FGA tasks (Fig. 7a; FGA 8, 9, and 10), (2) 5 FGA tasks for which significant differences were typically observed between patients and controls (Fig. 7b; FGA 3, 4, 8, 9, and 10), and (3) across all 8 tasks for which gait cycles were extracted (Fig. 7c), with the score scaled (see “Methods”) over a range from 0 (most abnormal) to 100 (normal).

Figure 7 illustrates that all three of these computations yielded similar results, with healthy controls scoring closest to 100%, followed by preoperative patients, chronic postoperative patients, and then acute postoperative patients. Further, controls were significantly different than patients at all 3 time points (*p* < 0.001) regardless of the computation. More specifically, kinematic-based scores focused on the most challenging tasks resulted in more prominent probability peaks for our postoperative patients (Fig. 7a,b; top right panels). By comparison, using all tasks resulted in slightly tighter probability distribution for control subjects (Fig. 7c). Thus, our analysis highlights the robustness of a computation based on these three specific kinematic measures (i.e., task completion speed, vertical head acceleration variability, and vertical head acceleration range of motion), Further, these results highlight the potential utility of focusing on a directed subset of the standard FGA (e.g., a "mini-FGA” comprising the 3 most challenging FGA tasks 8, 9, and 10) in the assessment of the vestibular function. To develop the optimal approach to generate a single “kinematic score” from head kinematic data obtained during the FGA, however, further algorithm development and testing utilizing a larger data base is required and is a very interesting direction for future work in this field.

Finally, we note that in general, there were no correlations between our 3 VOR clinical measures (i.e., VOR gain, VOR asymmetry, and VOR time constant) and the parameters calculated by quantifying head movement during the FGA tasks – with one notable exception. Specifically, we considered correlations between each of these three VOR measures and 16 kinematic measures used to quantify each of the specific FGA tasks, at three time points (i.e., preoperative, postoperative, or chronic). Only one of our VOR parameters (VOR time constant) showed a correlation with head movement parameters and this was only for one specific FGA task as the VOR time constant was correlated with speed measures (cycle duration and task completion) during “gait with eyes closed” task in our chronic postoperative patients (*p* < 0.01).

## Discussion

In this study, we quantified head kinematics during specific tasks of the Functional Gait Assessment (FGA) in patients with VS before and following unilateral vestibular neurectomy and healthy control subjects. Overall, we found that head movement kinematics were significantly altered as a result of a peripheral vestibular loss. Specifically, we identified important distinctions between patients and healthy controls, as well as between patients at different time points before and after surgery. First, we found significant differences in patient kinematics for some of the more challenging FGA tasks—at all 3 time points—when compared to age-matched healthy controls (i.e., ‘gait with a narrow base of support’, ‘gait with eyes closed’, ‘ambulating backwards’, and ‘steps’ task). Second, the comparison of patient head movement kinematics before surgery and sub-acute post-op revealed specific and substantive changes. Finally, our results established that patients head movement kinematics were remarkably comparable before and six months following surgery. Below we consider these observations in more detail, as well as the implications of our results in relation to neural mechanisms that mediate vestibular compensation.

A key finding of this study is that head motion kinematics in VS patients differ from those in normal subjects before surgery as well as in the sub-acute and chronic states after tumor resection and vestibular nerve section. Most notably, the range of motion and variability of head movements along the vertical axis were reduced in VS patients in all three clinical states. These differences were least pronounced in the pre-op patients as they were observed only on the most challenging conditions, were most pronounced in the sub-acute post-op state, and then subsided at the > 6-month state in which they approached pre-op values. Given that walking tasks underlie the FGA, it is logical that the vertical axis would be most affected by the vestibular state since vertical linear accelerations are large during these gait tasks, particularly when the heel strikes the ground (e.g.,^10,11^). This combination of findings for vertical axis head motion (i.e., reduced range of motion and reduced variability) suggests that patients with vestibular damage compensate in part by reducing the amplitude of their head movements, which in turn reduces variability (since variability scales with amplitude). Decreased variability cannot be directly due to peripheral vestibular changes since the reduction (due to the VS) or loss (due to neurectomy) of vestibular afference from one ear should actually increase signal variability and in turn, degrade the central signal-to-noise ratio (because the redundancy between the two ears is reduced or lost (reviewed in^12^). Indeed, this is consistent with the findings of single-unit recording experiments at the first central stage of processing in the vestibular nuclei of rhesus monkeys. Specifically, following a peripheral vestibular loss, individual central neurons show increased variability resulting in a significantly degraded signal/noise ratio than that observed in neurons in normal animals^13^. Thus, we conclude that the decreased variability observed in our patients is likely not the result of a change in central neuronal variability. Instead, we propose that the principal effect of vestibular nerve damage or loss on head movement kinematics is that subjects reduce the amplitude of head motion (principally along the vertical axis), and this amplitude reduction secondarily reduces head motion variability^14^.

To date, studies comparing vestibular schwannoma patients before and after vestibular neurectomy generally quantified impairments using clinical measures, primarily the VOR but also vestibular evoked myogenic potentials (e.g.,^15–18^), and interestingly we found that the widely used VOR measurements were generally not correlated with the head movement parameters we calculated. As noted above, our present quantification of head motion kinematics revealed that pre-op vestibular schwannoma patients could be distinguished from healthy controls during challenging FGA tests. Specifically, patients demonstrated increased lateral head acceleration and slower performance during the gait with a narrow base of support’ task—also known as tandem walking^19^. They were also slower and demonstrated a decreased range of motion and variability in the vertical axis during the ‘gait with eyes closed’ (task 8), ‘walking backward’ (task 9), and ‘steps’ (task 10). Thus, our approach appears to provide a more sensitive measure than those used in prior studies reporting no difference between controls and pre-op vestibular schwannoma patients during these same tasks^20^. In contrast, our pre-op vestibular schwannoma patients were indistinguishable from healthy controls during less challenging FGA tests. This latter observation is consistent with previous studies reporting normal gait during standard walking based on other measures (e.g., force sensors^20^, video^21^, and subjective scoring^22^).

We further note that we could not specifically isolate the effects of deafferentation in this study since the earliest post-op data was acquired 6 weeks after vestibular neurectomy. Substantial compensation had certainly occurred in this interval, but even with these caveats, we were able to identify significant differences between head kinematics in the pre-op and sub-acute post-op states. The most prominent change was in the ‘walking with horizontal head turns’ (task 3), where the interaural motion amplitude and variability were smaller in the sub-acute post-op state. Large angular velocities are generated during voluntary yaw head rotations, so it is unsurprising that the gait task that employs this type of voluntary head motion would be the most abnormal after neurectomy. While we did not explicitly measure gaze, we speculate that subjects did their best to align their gaze direction with their voluntary head movement as instructed and that patients experienced poorer gaze stability during this task (e.g.,^23,24^). Indeed, recent studies have reported smaller and fewer yaw horizontal head turns during self-paced walking in patients after resection of vestibular schwannoma^25,26^. Again, we suggest that the principal response to deafferentation is the reduction in head movement amplitude and that reduced variability follows, rather than the reduction in variability being a direct effect of deafferentation. Furthermore, while vertical axis movements were the most abnormal prior to VS surgery, deafferentation had only minor effects on vertical head motion. While reason for this is uncertain, it is possible that a partial reduction in vestibular function, which was present in most pre-op VS patients (as evidenced by abnormal pre-op VOR responses) may be adequate to make this type of head motion highly provocative and therefore lead patients to suppress the range of vertical axis head motion.

Importantly, as noted above, we cannot disentangle early compensation effects from deafferentation effects in this study since the earliest post-op measurements were made after 6 weeks – patients with acute unilateral vestibular dysfunction generally show the most significant compensation within the first week^27–30^. The relative subtle differences between pre-op and sub-acute post-op results, however, suggest that prominent compensation occurred during this sub-acute period since much larger changes in head movements would be predicted after unilateral deafferentation. After 6 weeks, compensatory changes continued, as evidenced by the improved head movement characteristics in the chronic post-op data compared to the sub-acute post-op data. Head movements in the chronically deafferented VS patients were very similar to the movements made by these patients prior to VS surgery and remained different than normal. There are clear constraints on the efficacy of compensation in terms of its effects on head kinematics, therefore, as the optimally compensated patients (i.e. chronic post-op) with unilateral deafferentation are essentially the same as the (presumably well-compensated) pre-op patients who rarely have complete destruction of the vestibular nerve by the tumor. The presence of vestibular damage, rather than its extent, therefore, appears to be the factor that sets the upper limit for recovery of normative head motion in these patients.

Thus taken together, our results indicate that patients show a reduction in both head motion amplitude and variability, even prior to surgery as a result of the presence of the tumor. We speculate that these changes in head motion strategy are potentially maladaptive for vestibular compensation. First, as noted above, a reduction in behavioral variability can be counterproductive for motor learning and adaptation (reviewed in^31^). Further, the observed reduction in head motion amplitude would result in a decrease of vestibular as well as extra-vestibular information during natural behaviors. Single unit recording experiments in monkeys have shown that compensation is mediated, in part, by the unmasking of extravestibular inputs (e.g., proprioceptive and motor commands) at the level of the vestibular nuclei following a peripheral vestibular loss^32–34^. This extravestibular sensory and motor substitution provides a concrete neural substrate for improvements in self-motion perception^13^ and the efficacy of rehabilitation programs focusing on enhancing sensory reweighting as soon as possible after vestibular loss (reviewed in^35^ and^36^). Thus, given that patients already demonstrated reduced head movement before surgery, we speculate that this strategy is maladaptive in that it constrains an individual’s ability to improve following surgery. We suggest that objective measures such as head movement range of motion and variability during voluntary movements such as gait provide information vital for evaluating patient strategies before as well as after surgery that can inform interventions to improve outcomes.

Finally, it is noteworthy that while standard clinical tests focusing on vestibular-mediated eye movements do not correlate well with clinical status, test batteries such as the FGA do provide meaningful information about postural control and fall risk. However, these common tests are inherently limited by observer-based grades that are captured by integer values. Our present data suggest that the more complex and subtle data set provided by small six-dimensional sensors on the head (and potentially trunk) will prove to correlate more strongly with postural control and fall risk than the current 0–30 score generated in standard FGA observer scoring. Notably, our results suggest that objective kinematic measures can reveal postoperative changes that cannot be identified by the conventional 0–30 FGA scoring. Furthermore, we speculate that data measured by sensors will propel balance test batteries to become shorter, more refined, and more predictive of clinical status. In this regard, our study provides an example of how changes in peripheral vestibular function and central compensation can be quantified by measuring head kinematics. Current availability of wearable sensors as well as their accuracy for measuring gait parameters^37–40^ make them suitable for augmenting the information during clinical gait assessments such as FGA. Also, given the importance of head motion in both understanding vestibular processing^41–44^ and diagnosing and treating vestibular disorders^15,22,25,26,45–51^, we expect that this field will expand in importance over the next few years and will eventually become an integral component of clinical vestibular medicine.

## Methods

This study was implemented in accordance with the Declaration of Helsinki, was approved by the Massachusetts Eye and Ear Infirmary (MEEI) IRB. All subjects were provided informed consent for this study.

### Subjects

Our study comprised two groups: patients and age-matched healthy control subjects. The first study group consisted of 23 patients (13 female and 10 male) with a diagnosis of unilateral vestibular schwannoma (VS) who were to undergo surgical resection of their tumor at MEE via suboccipital craniotomy and retrosigmoid approach with complete sectioning of the vestibular nerve (11 and 12 on the left versus right side). This study group was tested before surgery (“pre-op”) and then 6 weeks after surgery (“sub-acute post-op”) and > 6 months after surgery (“chronic post-op”). VS patients had no other history of neurologic or otologic disease and patients with Neurofibromatosis 2 (NF2 were excluded. The second group included 21 age-, gender-matched controls (8 female and 13 male) who did not have any history of otologic or neurologic disease.

### Sinusoidal vertical-axis rotation

During sinusoidal vertical axis rotation, subjects were seated upright and were rotated about an earth-vertical yaw axis at seven frequencies between 0.01 and 1.0 Hz, 40 deg/s peak velocity. Eye movements were recorded using an eye-tracking goggle system (Neuro Kinetics, Inc), and head movements were assumed to reflect chair motion since the head was immobilized relative to the chair. Eye movement traces were desaccaded with an automated program, and the slow phases were fit to sine with minimum mean-squared-error, then the gain, phase, and bias values were calculated across frequencies. The VOR gain and time constant (TC), and asymmetry were calculated as previously described in^52^.

### Functional gait assessment (FGA)

The FGA is used to assess postural stability during ten specific walking tasks including (1) gait on a level surface, (2) change in gait speed, (3) gait with horizontal head turns, (4) gait with vertical head turns, (5) gait and pivot turn, (6) step over obstacle, (7) gait with narrow base of support, (8) gait with eyes closed, (9) ambulating backwards, and (10) steps (Fig. 1a). The FGA adds three relatively challenging tasks (i.e., tasks 7 to 9) to the Dynamic Gait Index (DGI), to reduce the ceiling effect in the vestibular patients. A clinical expert scored each task between 0—3 points and the total FGA score is the sum of these ten individual scores (Table 1).

To quantify head movement kinematics during the FGA, we recorded each subject’s head movements in six dimensions using a micro-electromechanical system (MEMS) module (iNEMO platform, STEVAL-MKI062V2, STMicroelectronics), which comprises three linear accelerometers (recording linear accelerations up to 8 g along the fore-aft, interaural, and vertical axes) and was augmented by a STEVAL-MKI107V2 three axis gyroscope (angular velocity up to 2000dps about pitch, roll, and yaw; Fig. 1b). Note, this same MEMs based platform has been used in previous studies of normal human subjects and patients^41,43,46^. The data from the six sensors were sampled at 100 Hz and recorded wirelessly on a microSD card. The MEMS module, the battery, and the microSD card were grouped in an extremely light (64 g) and small (35 × 35x15 mm) enclosure. This enclosure was comfortably attached to the top of the subject’s head (see ref.^41^). The plane spanned by the fore-aft, and the interaural axes of the MEMS module were set parallel to the subject’s Frankfurt plane (i.e., the plane passing through the inferior margin of the orbit to the external auditory meatus).

### Data analysis

Head movement data during FGA were filtered with a low-pass filter with 25 Hz cutoff frequency. Then, for the 8 out of the ten tasks (i.e., tasks 1–4,6,8–10 in Fig. 1a, Table 1) during which it was possible to detect individual gait cycles, we identified each gait cycle of each task based on the clear peaks in the vertical head acceleration signal, which corresponded to each foot's heel-strike (Fig. 1c). For each of these tasks, we computed measures of gait speed, symmetry, variability, and range of motion. First, we computed two measures of *speed*, namely the (1) ‘completion speed’, computed distance accomplished for each task divided by the time of completion, and (2) ‘step speed’, estimated as the average number of steps per second during the task. We also computed two measures of *asymmetry* using head acceleration measurements: (1) ‘step time asymmetry’, which was estimated from the time intervals between vertical acceleration peaks for each leg's heel strike was calculated and (2) ‘step length asymmetry’, estimated as the integration of the positive vertical accelerations following heel strike of each leg. Asymmetry was calculated by (1) dividing the deafferented side value by the intact side value (blue versus red shaded regions, Fig. 1c) for the patients and (2) dividing the left side value by the right-side value for control subjects^53^. *Variability* and *range of motion* measures were computed by first normalizing all gait cycle durations. Variability was then quantified for each of the three linear acceleration (fore-aft, interaural, and vertical) and three angular velocities (roll, pitch, and yaw) axes of head motion acceleration) as the average of the standard deviation of the signal across gait cycles in a given task (Fig. 1d; denoted by green shaded bands). Similarly, the range of motion during each task was quantified by computing the difference in the maximum versus minimum values achieved on average throughout the gait cycle in a given task (Fig. 1d; denoted by orange arrow). This analysis was performed for each of the six dimensions of head motion.

For the remaining two tasks (e.g., tasks 5 and 7; Fig. 1a and Table 1), during which the gait cycle was difficult to detect, we computed other specific measures. Specifically, for task 5 (‘gait and pivot turn’) we calculated the (1) peak yaw velocity obtained during the pivot turn and (2) post-pivot head acceleration, calculated as the root mean square of linear accelerations along all axes for a two-second interval following the of the pivot turn. For task 7 (‘gait with a narrow base of support’) task we quantified (1) subject speed (i.e., distance achieved during the task/time required for completion) and (2) head acceleration, again calculated as the root mean square of linear accelerations along all axes (i.e. fore-aft, interaural, and vertical).

In order to compare between control and patient groups, we used a permutation test (independent sample). In order to compare measures from our patients tested at different time points (before, 6 weeks after, and > 6 months after surgery), we used a paired sample permutation test with a significance level of 0.05. Kinematic score was computed based on three most informative gait measure: (1) task completion speed, (2) vertical head acceleration variability, and (3) vertical head acceleration range of motion. First, each of three kinematic measure during each FGA task was normalized by a linear transformation of mean ± 2SD to a number between 0 and 100 (i.e., normalized mean = 50 and normalized SD = 25). Numbers outside the 0–100 range were then projected to closest number within this range (i.e., either 0 or 100). The average of three normalized numbers across all selected FGA tasks was used as the kinematic score. All data processing and statistical tests were performed using a custom script in MATLAB (The MathWorks, Inc., Natick, Massachusetts, United States). Throughout, values are expressed as mean ± SEM.

## Data Availability

The data that support the findings of this study are available from the corresponding author, K.E.C, upon reasonable request.
